# Off-Label but On-Target: Sacral Neuromodulation for Neurogenic Bladder Dysfunction

**DOI:** 10.1007/s11934-025-01280-6

**Published:** 2025-06-23

**Authors:** Keianna Vogel, Nissrine Nakib

**Affiliations:** Department of Urology, University of Minnesota, Minneapolis, 420 Delaware St SE, MMC 394, MN 55455 USA

**Keywords:** Sacral neuromodulation, Neurogenic bladder, Lower urinary tract dysfunction

## Abstract

**Purpose of Review:**

Neurogenic bladder dysfunction (NBD) is a frequent complication of neurological diseases including Parkinson’s disease, multiple sclerosis, spinal cord injury, and spina bifida. Managing NBD remains challenging, especially for patients who fail to respond to standard therapies. Sacral neuromodulation (SNM), though FDA-approved for non-neurogenic conditions, is increasingly explored off-label in neurogenic populations.

**Recent Findings:**

Recent studies demonstrate that SNM can improve lower urinary tract symptoms, bladder storage, and emptying in select NBD patients. Small case series and retrospective reviews suggest reduced catheterization and medication use. Technological advances, such as closed-loop systems and MRI-compatible devices, are broadening SNM’s clinical potential.

**Summary:**

SNM is emerging as a valuable, minimally invasive treatment for neurogenic bladder dysfunction. Careful patient selection and understanding of underlying pathophysiology are essential for success. As evidence grows and device innovations continue, SNM could reshape management strategies for patients with neurogenic bladder.

## Introduction

Neurogenic bladder dysfunction (NBD), typically linked to neurological diseases such as Parkinson’s (PD), multiple sclerosis (MS), and spinal cord injury (SCI), presents complex treatment challenges. Sacral neuromodulation (SNM), though FDA-approved for conditions like overactive bladder (OAB), non-obstructive urinary retention, and fecal incontinence (FI), is increasingly used off-label in patients with NBD — prompting important clinical and scientific questions. This review explores how SNM works, its evolving indications, and the diagnostic complexities surrounding neurogenic bladder. We propose that symptoms often attributed solely to neurological disease may stem from a broader interplay of factors, and that SNM could offer meaningful relief for carefully selected patients.

This review synthesizes recent literature (2019–2024) to evaluate the efficacy of sacral neuromodulation (SNM) in the treatment of neurogenic bladder. We trace the historical development of SNM, explore its underlying physiological mechanisms, and examine current clinical indications. We also address diagnostic complexities that may obscure the identification of true neurogenic bladder and highlight emerging controversies and priorities for future research.

### History of Sacral Neuromodulation

The foundation of sacral neuromodulation (SNM) was laid in the early 1990s. In 1992, Dr. Steven Siegel introduced a staged technique that remains central to contemporary SNM practice. This approach—beginning with test stimulation in the sacral foramen to elicit motor responses, followed by temporary electrode placement, and culminating in permanent implantation for responders—enhanced both patient selection and therapeutic outcomes [1]. SNM devices received FDA approval in 1997. Initially developed for idiopathic overactive bladder (OAB), the technique’s clinical success has led to broader applications in patients with suspected neurogenic bladder. However, the pathophysiology of lower urinary tract symptoms (LUTS) in neurogenic populations is complex. Symptoms traditionally attributed to neurological dysfunction may also arise from secondary factors such as detrusor overactivity, medication effects, or maladaptive voiding behaviors.

### FDA-Approved Indications

Currently, the U.S. Food and Drug Administration (FDA) has approved SNM for the treatment of overactive bladder, non-obstructive, non-neurogenic urinary retention, and fecal incontinence. These approvals are supported by randomized controlled trials demonstrating SNM’s efficacy in patients who have not responded to conservative therapies [2].

### Mechanism of Action

The precise mechanism by which SNM exerts its therapeutic effects remains incompletely understood. The leading hypothesis suggests that electrical stimulation of the sacral nerves influences afferent and efferent signaling between the bladder, pelvic floor, and central nervous system. This neuromodulation is thought to recalibrate dysfunctional reflexes and neural circuits associated with lower urinary tract symptoms.

In patients with spinal cord injuries, however, the efficacy of SNM is often reduced. This diminished response likely reflects disruption of the neural pathways connecting the sacral spinal cord to supraspinal centers—pathways that are critical for effective neuromodulation. As a result, individuals with complete SCI may not experience the same therapeutic benefit as those with preserved neural continuity [3].

### Expanding Indications and Off-Label Applications

Beyond its FDA-approved indications, sacral neuromodulation (SNM) has been increasingly explored in a variety of off-label contexts for patients with neurological disease. This section reviews current evidence for SNM in Parkinson’s disease (PD), multiple sclerosis (MS), spina bifida, and the broader category of neurogenic bladder (NB).

#### Parkinson’s Disease (PD)

Lower urinary tract dysfunction is common in patients with PD and is thought to arise from degeneration of dopaminergic pathways involved in autonomic bladder control. A critical mechanism in normal voiding is the coordination between the sympathetic and parasympathetic nervous systems. In PD, bradykinesia may impair this coordination, leading to detrusor contractions against a partially closed sphincter. SNM is hypothesized to help restore synchronized autonomic activity, thereby improving bladder emptying and reducing post-void residual volumes.

A retrospective study of twenty-two PD patients with overactive bladder (OAB) symptoms reported that 82% of those who completed the SNM test phase proceeded to permanent implantation, and 68% of implanted patients were able to discontinue OAB medications [4]. Another study evaluating long-term outcomes in PD patients with neurogenic OAB supported SNM as an effective treatment option in this population. However, the study also noted that urodynamic findings suggestive of bladder outlet obstruction may predict treatment failure, emphasizing the need for careful patient selection [5].

#### Multiple Sclerosis (MS)Multiple Sclerosis (MS)

SNM has demonstrated promising results in patients with MS, particularly those with stable disease and minimal spinal cord involvement. Studies show improvements in urgency, frequency, and urinary retention. However, the progressive demyelination and neurodegeneration characteristic of MS may limit long-term SNM efficacy, as declining neural conduction reduces the bladder’s responsiveness to stimulation [6, 7].

While conventional management includes medications, behavioral therapies, and intermittent catheterization, these options often provide incomplete relief and may cause side effects such as dry mouth or recurrent urinary tract infections. SNM offers a targeted, non-systemic alternative that avoids these adverse effects. Evidence suggests that patients with relapsing-remitting MS or incomplete spinal involvement achieve better outcomes than those with progressive forms of the disease. Future research should aim to stratify patients by lesion location and disease severity to optimize candidate selection.

A retrospective review of eighteen MS patients undergoing SNM showed significant improvement in urinary symptoms. The average Urogenital Distress Inventory (UDI-6) score dropped from 56.6 pre-implantation to 25.2 post-implantation (*p* < 0.0001). Similarly, the Incontinence Impact Questionnaire (IIQ-7) score decreased from 59.0 to 22.2 (*p* < 0.001), and the Global Response Assessment (GRA) showed an average score of 6, indicating “moderately improved” status [8].

A separate case series of six MS patients with severe urinary incontinence demonstrated improvement using the Michigan Incontinence Symptom Index (M-ISI), with scores declining from 28 pre-operatively to 6 post-implantation. All six patients reported subjective improvements in quality of life [9]. Both studies were limited by small sample sizes, short follow-up durations, and lack of stratification by disease severity—highlighting the need for larger, controlled studies to determine long-term effectiveness and refine patient selection.

#### Spina Bifida

Patients with spina bifida frequently experience significant lower urinary tract dysfunction, including incontinence, impaired bladder emptying, and recurrent urinary tract infections. Conventional treatments include clean intermittent catheterization (CIC), medications, and, in some cases, bladder augmentation or urinary diversion. These strategies, however, are often limited by adherence issues and long-term complications.

Recent studies suggest that SNM may improve continence and reduce the need for invasive interventions in ambulatory spina bifida patients. Improvements in bladder storage and voiding function have been observed, with reported increases in bladder compliance, reductions in detrusor overactivity, and fewer incontinence episodes [10]. SNM offers a less invasive alternative that modulates sacral reflex pathways to enhance both bladder and bowel function.

Furthermore, earlier intervention with SNM in younger patients may improve long-term outcomes and delay or even prevent the need for more aggressive surgical procedures. Despite its potential, patient selection remains critical. SNM appears to be most effective in patients with preserved sacral reflexes and incomplete neurological deficits. Anatomic challenges such as pelvic deformities may complicate supine positioning and optimal lead placement under fluoroscopy. More robust, long-term studies are needed to evaluate sustained benefits and address the unique programming considerations in this population.

#### eurogenic Bladder (NB)

The International Continence Society defines neurogenic bladder as bladder dysfunction resulting from central or peripheral nervous system pathology. SNM may benefit this population by modulating afferent nerves to restore reflex voiding circuits, improve bladder sensation, inhibit detrusor overactivity, and re-establish coordinated detrusor-sphincter activity.

Neurogenic bladder is highly prevalent: 37–72% of PD patients, 40–90% of those with MS, 15% of stroke patients, and 70–84% of individuals with spinal cord injury are affected [11]. Recent studies involving patients with neurogenic lower urinary tract dysfunction (NLUTD) due to MS, SCI, PD, stroke, and spina bifida report that 52–72% who undergo SNM proceed to permanent lead placement, with many reporting subjective improvements in quality of life (Table 1) [12–14].

Despite this growing evidence base, SNM is not yet formally indicated for NLUTD. Barriers to FDA approval include the logistical and financial challenges of conducting large-scale randomized controlled trials in heterogeneous populations, as well as the difficulty of defining uniform inclusion criteria and outcome measures across varying neurological etiologies.

Nevertheless, the high prevalence of neurogenic bladder and the limitations of current therapies suggest a substantial unmet need. Expanding SNM indications in appropriately selected patients could improve symptom control, bladder function, and quality of life while reducing reliance on more invasive or less effective treatment modalities.

These emerging applications of SNM across diverse neurological conditions underscore its potential as a valuable, minimally invasive treatment for neurogenic bladder dysfunction. While current evidence—though limited by small sample sizes and study heterogeneity—suggests meaningful symptomatic improvement in carefully selected patients, broader clinical adoption remains constrained by the absence of FDA approval and a lack of high-quality randomized trials. Moving forward, collaborative multicenter studies with standardized protocols will be essential to validate SNM’s efficacy, identify predictors of response, and inform evidence-based guidelines. As our understanding of disease-specific pathophysiology deepens, so too will opportunities to personalize neuromodulation therapies and expand access to this promising intervention for underserved patient populations.

## Future Directions

The future of sacral neuromodulation (SNM) for neurogenic bladder is promising, with several technological and clinical advancements poised to expand its utility. One emerging frontier is personalized neuromodulation, particularly the development of closed-loop SNM systems. These systems dynamically adjust stimulation parameters in real time based on neural or physiologic feedback. Unlike traditional fixed stimulation protocols, adaptive systems have the potential to improve long-term efficacy and reduce therapy failure by tailoring treatment to individual bladder activity patterns [15].

Early intervention in acute spinal cord injury represents another critical research area. Preliminary studies suggest that initiating SNM therapy shortly after spinal trauma may help preserve neural circuitry and prevent secondary bladder dysfunction. By intervening prior to irreversible neural damage, SNM could reduce long-term dependence on catheterization and minimize complications such as detrusor sphincter dyssynergia, thereby improving functional outcomes [16].

As clinical evidence continues to accumulate, there is growing support for expanding SNM indications and pursuing formal regulatory approval for neurogenic bladder. While SNM is not currently FDA-approved for this indication, mounting data indicate significant benefit in appropriately selected patient populations. To achieve broader adoption, large-scale, multicenter clinical trials are needed to establish standardized treatment protocols, refine patient selection criteria, and provide the regulatory framework necessary for approval [12, 17, 18].

Beyond standalone therapy, combination strategies are being investigated to augment SNM outcomes. These include concurrent use with pharmacologic agents, botulinum toxin injections, and regenerative medicine approaches. An especially exciting avenue involves combining SNM with urothelial cell-based therapies aimed at promoting nerve regeneration in neurogenic bladder, with the potential to reverse or mitigate disease progression [15].

The field is also witnessing the rise of minimally invasive and wearable neuromodulation technologies. Innovations in bioelectronic medicine have led to the development of external or injectable SNM systems that could provide similar benefits to traditional implants while reducing surgical risks. These technologies may broaden access to SNM, particularly for patients who are not candidates for conventional implantation.

Enhancing device longevity and reliability remains a priority. Improvements in battery technology—such as longer-lasting or rechargeable implantable pulse generators—are crucial for reducing the frequency of revision surgeries and improving patient adherence [1].These developments will help ensure that SNM remains a durable and cost-effective long-term therapy.

In parallel, advances in needle placement accuracy may significantly enhance SNM efficacy. Improved techniques and imaging guidance can facilitate more consistent neural targeting, reduce lead migration, and minimize complications—especially in patients with challenging anatomy or positioning constraints.

Lastly, the integration of telemedicine and remote monitoring into SNM care models holds transformative potential. Remote programming and monitoring platforms could enable clinicians to track outcomes, adjust therapy parameters in real time, and manage care more efficiently. These capabilities may improve treatment adherence, reduce the burden of follow-up visits, and enhance access for patients in remote or underserved regions.

Together, these innovations signal a transformative era for sacral neuromodulation in the management of neurogenic bladder. Advances in personalized therapy, device design, surgical technique, and remote monitoring promise to expand access, improve long-term outcomes, and optimize patient-centered care. As research continues to evolve, interdisciplinary collaboration and robust clinical trials will be essential to translate these developments into routine practice and secure the broader adoption of SNM for this complex and underserved patient population.

## Conclusion

Sacral neuromodulation represents a promising and increasingly utilized intervention for patients with presumed neurogenic bladder, particularly those exhibiting functional rather than strictly structural or etiological dysfunction. While the exact mechanism of action remains incompletely understood, SNM is believed to restore bladder control by modulating aberrant neural pathways between the sacral nerves and central nervous system. Its application has shown encouraging results across a spectrum of neurological conditions including Parkinson’s disease, multiple sclerosis, spinal cord injury, and spina bifida.

A key insight emerging from recent literature is the importance of refining patient selection to improve treatment outcomes and broaden access to SNM. This entails a shift from rigid diagnostic categories to a more nuanced evaluation of patients based on symptom profiles, urodynamic findings, and responsiveness to prior therapies. In other words, optimal candidates should be identified through functional assessment rather than neurologic diagnosis alone, recognizing that similar lower urinary tract symptoms may arise from heterogeneous etiologies, such as such as detrusor overactivity, medication effects, or maladaptive voiding behaviors.

An additional consideration in this population is the method of SNM trialing. While the staged implant approach remains the predominant method in most studies, percutaneous nerve evaluation (PNE) has also been utilized in select NGB cohorts. Existing literature indicates that both PNE and staged trials may be feasible in this context, though their relative diagnostic and predictive value in neurogenic populations remains underexplored. PNE offers the advantages of reduced invasiveness and lower upfront cost but may be limited by patient mobility, lead migration, or challenges in sensory feedback, factors particularly relevant in neurologically impaired individuals. Staged trials, by contrast, offer more durable lead placement and may better accommodate the complex and evolving symptomatology seen in NGB patients. A nuanced understanding of patient-specific factors, such as motor function, cognitive status, and bladder phenotype, should guide trial method selection in future studies.

As technology evolves and experience with SNM deepens, future research must prioritize stratified, multicenter trials to better define functional predictors of response and optimize care pathways based on functional profiles to enable a more personalized application of SNM. With continued innovation in devices, programming, and care delivery, SNM holds significant potential to transform the management of neurogenic bladder and improve quality of life for a historically underserved patient population.

**Table 1:** Table 1. Recent Studies Assessing Sacral Neuromodulation in Neurogenic Bladder Dysfunction

Reference	Year	Study Type	Underlying Disease	*N*	Definition Of Success	Outcome
Martin et al. [4]	2022	Retrospective cohort	Parkinson’s Disease	34	Test phase success: >50% improvement in urinary symptoms	82%; 28/34 progressed to permanent implantation
Chen et al. [8]	2022	Retrospective cohort	Multiple Sclerosis	18	Scores on UDI-6, IIQ-7, and GRA	UDI-6 improved from 54.6 to 22.6 with 15/22 reporting improvement. IIQ-7 improved from 60.7 to 20.8
Thys et al. [9]	2023	Case series	Multiple Sclerosis	6	M-ISI	Improvement in M-ISI from 28 to 6
Buford et al. [13]	2024	Retrospective cohort	NLUTD	82	Test phase success: >50% improvement in urinary symptoms	72%; 59/82 progressed to permanent lead implantation
Chen et al. [10]	2021	Retrospective cohort	Spina Bifida	29	Test phase success: >50% improvement in urinary symptoms	55%; 16/29 progressed to permanent lead implantation (21/29 with > 50% symptom improvement)
Masood et al. [14]	2021	Retrospective cohort	NLUTD	152	Test phase success: >50% improvement in urinary symptoms	68%; 104/152 progressed to lead implantation
Liechti et al. [12]	2022	Randomized Controlled Trial	NLUTD	124	Test phase success: >50% improvement in urinary symptoms	52%; 60/124 progressed to lead implantation

UDI-6– Urogenital Distress Inventory, IIQ-7– Incontinent Impact Questionnaire, GRA– Global Response Assessment, M-ISI– Michigan Incontinence Symptom Index, NLUTD– Neurogenic Lower Urinary Tract Dysfunction

## Key References

- Liechti MD, van der Lely S, Knüpfer SC, Abt D, Kiss B, Leitner L, et al. Sacral neuromodulation for neurogenic lower urinary tract dysfunction. NEJM Evid. 2022;1(11):EVIDoa2200071. 10.1056/EVIDoa2200071.

- Randomized controlled trial assessing sacral neuromodulation in neurogenic lower urinary tract dysfunction.

- Buford K, Eisner H, Vollstedt A, Friedman B, Gilleran J, Zwaans BMM, et al. Implantable neuromodulation for neurogenic lower urinary tract dysfunction: a single-institution retrospective study. Int Neurourol J. 2024;28(4):278–284. 10.5213/inj.2448144.122.

- Largest recent retrospective review validating use of sacral neuromodulation in neurogenic lower urinary tract dysfunction

- Spilotros M, Gerbasi S, Lasorsa F, de Rienzo G, Balducci L, Ditonno P, et al. Sacral Neuromodulation: Device Improvement and Current Applications in Urology. Medicina (Kaunas). 2024;60(3):509. 10.3390/medicina60030509.

- Reviews the technical advances and expanding clinical uses of sacral neuromodulation offering an understanding of the contemporary role of sacral neuromodulation.

## Data Availability

No datasets were generated or analysed during the current study.
